# Synthesis of Co_2_FeGe Heusler alloy nanoparticles and catalysis for selective hydrogenation of propyne†

**DOI:** 10.1039/d1ra02884g

**Published:** 2021-05-19

**Authors:** Takayuki Kojima, Yuki Nakaya, Hyungwon Ham, Satoshi Kameoka, Shinya Furukawa

**Affiliations:** Frontier Research Institute for Interdisciplinary Sciences, Tohoku University Sendai Japan; Institute of Multidisciplinary Research for Advanced Materials, Tohoku University Sendai Japan; Institute for Catalysis, Hokkaido University Sapporo Japan furukawa@cat.hokudai.ac.jp

## Abstract

Although intermetallic compounds are attracting attention of catalysis researchers, ternary intermetallic catalysts have scarcely been investigated due the difficulty of synthesizing supported nanoparticles. In this study, we successfully synthesized SiO_2_ supported Co_2_FeGe Heuslar alloy nanoparticles. This catalyst exhibited high catalytic performance for selective hydrogenation of propyne by nano-sizing.

## Abstract 2

Supported nanoparticles of Co_2_FeGe Heusler alloy were successfully synthesized. The reaction rate per weight was increased 2000 times by nano-sizing.

## Graphical abstract

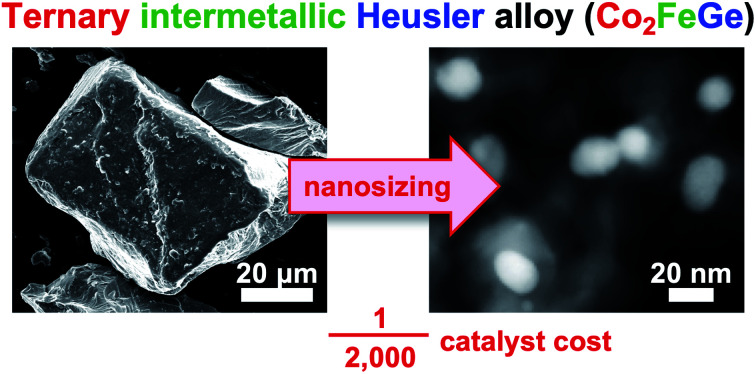

An alloy is a solid mixture of two or more metallic elements. It is typically categorized into a solid solution and an intermetallic compound. In the former, different metal atoms randomly occupy lattice points, and the available range of chemical compositions is wide. In the latter, different metal atoms occupy specific lattice points, forming an ordered structure, and chemical compositions available are restricted to integer ratios. Thus, intermetallic compounds have unique electronic structures and unique atomic ordered surfaces, which are completely different from those of pure metals and solid solutions, resulting in novel catalytic properties.^1–6^

Along with a recent increasing interest in intermetallic catalysts, many binary compounds have been investigated as catalysts thus far; however, ternary compounds have scarcely been reported as catalysts. The number of possible elemental sets forming intermetallic compounds is much larger in ternary systems than binary ones.^7^ In addition, novel properties originating from synergy among different elements are more likely in ternary than binary systems; for example, in the La(Co or Ru)Si catalyst for ammonia synthesis, the hydrogen storage ability, the electride property, and the electron transfer from La to the active element (Co or Ru) are believed to play key roles.^8,9^ Therefore, the discovery of various new catalysts is expected in ternary systems.

Heusler alloys are a group of ternary intermetallic compounds described by X_2_YZ with L2_1_ structure (body-centered cubic basis) typically consisting of 8–12, 3–8, and 13–15 group elements for X, Y, and Z, respectively. This intermetallic group is popular as magnetic, thermoelectric, shape memory and topological materials while we have opened its new function as catalysts.^10–14^ For selective hydrogenation of alkynes, Co_2_FeGe Heusler alloy showed intrinsically high alkene selectivity; that is, it selectively hydrogenated alkynes but hardly hydrogenated alkenes even for hydrogenation of alkene reactants without alkynes.^11^ In addition, the systematic control of catalytic properties by elemental substitution (Co_2_Mn_*x*_Fe_1−*x*_Ga_*y*_Ge_1−*y*_) was demonstrated. However, these catalysts were unsupported micron-sized powders with low surface areas (<0.1 m^2^ g^−1^) synthesized by metallurgical process (arc melting, annealing, crushing), which were far from being of practical use. Thus, synthesis of Co_2_FeGe nanoparticles on solid supports, the standard form of catalysts assuring high activity per material cost, is desired.

To synthesize supported intermetallic nanoparticles, much effort is required to optimize the synthesis conditions, especially in ternary systems. For Heusler alloys, supported nanoparticles with sufficient quality (small average size with sharp distribution of sizes, small second phases, ordering into L2_1_ structure) have been reported only for Co_2_FeGa^15–18^ and Cu_2_NiSn,^19^ the former of which was not for catalysts but mainly for magnetic materials. Thus, synthesizing supported Co_2_FeGe nanoparticles with excellent catalytic properties for selective hydrogenation of alkynes is challenging. Nevertheless, we have achieved the synthesis of a variety of supported intermetallic nanoparticles,^4,6^ including those using three elements; for example, Pt_3_Fe_1−*x*_M_*x*_ (M = Co, Ni, Cu, Zn, Ga, In, Sn, Pb)^20^ and PtGa with deposition of Pb, In, or Sn.^21^

In what follows, we report the synthesis of SiO_2_ supported Co_2_FeGe nanoparticles and its catalytic properties for selective hydrogenation of propyne (C_3_H_4_). The Co-based catalysts were prepared by the pore-filling (co-)impregnation method using SiO_2_ as the support. Co(NO_3_)_3_·6H_2_O (Wako, 98%), Fe(NO_3_)_3_·9H_2_O (Sigma-Aldrich, 98%), (NH_4_)_2_GeF_6_ (Aldrich, 99.9%) were used as the metal precursors, and the Co loading was adjusted at 3 wt%. The metal precursors were precisely weighed and dissolved together in deionized water so that the Co : Fe : Ge atomic ratio was 1.8 : 1 : 1. A mixed aqueous solution of metal precursors was added dropwise to ground dried silica gel (CARiACT G-6, Fuji Silysia, *S*_BET_ = 673 m^2^ g^−1^) so that the solutions just filled the pores of the silica gel (volume of solution: 1.6 mL per gram of silica). The mixtures were sealed with a piece of plastic film and kept overnight at room temperature, followed by freeze-drying under vacuum at 0 °C and further drying overnight in an oven at 90 °C. The resulting powder was calcined in air at 500 °C for 1 h, then reduced under flowing H_2_ (0.1 MPa, 50 mL min^−1^) at 800 °C for 1 h.

Fig. 1 shows the powder X-ray diffraction (XRD) (Rigaku Ultima IV) pattern for the synthesized Co_2_FeGe/SiO_2_. Although tiny peaks of another phase (possibly CoGe) were detected by peak fitting, as shown in Fig. 1c and d, all visible peaks in Fig. 1a and b are assigned to Co_2_FeGe Heusler phase (L2_1_ structure). The peaks were broad, indicating the formation of nano-sized grains. The volume-weighted average grain size (*d*_XRD_) was roughly estimated to be 13 nm from the full width at half maximum (FWHM) of the 220 peak using the Scherrer equation:

1 *d*_XRD_ = *Kλ*/*W* cos *θ*

where *K*, *λ*, and *W* are the Scherrer constant, a wavelength, and a peak width, respectively; and 8/3π and FWHM were respectively adopted as *K* and *W* based on the assumption of spherical crystallites with lognormal size distribution.^22^ 111 and 200 superlattice peaks were certainly observed, meaning the formation of the ordered structure. These peaks are essentially very weak, because their intensities (*I*) are proportional to the square of the difference in the atomic scattering factors (*F*) of constituents, which are basically proportional to atomic numbers (*I*_111_ ∝ |*F*_Fe_ − *F*_Ge_|^2^, *I*_200_ ∝ |*F*_Co_ − (*F*_Fe_ + *F*_Ge_)/2|^2^). Considering the anomalous scattering factors,^23^ the Debye–Waller factors,^24,25^ the multiplicity factor, and the Lorentz-polarization factor, *I*_111_/*I*_220_ = 0.007 and *I*_200_/*I*_220_ = 0.004 are derived for the perfectly ordered case. The broadening also makes it difficult to detect the superlattice peaks. Thus, the detection of the superlattice peaks indicates that the L2_1_-ordered structure correctly formed even in nanograins. The degree of long-range order for Heusler alloys is evaluated typically by Webster's model using *S* and *α*^10,26,27^ as2
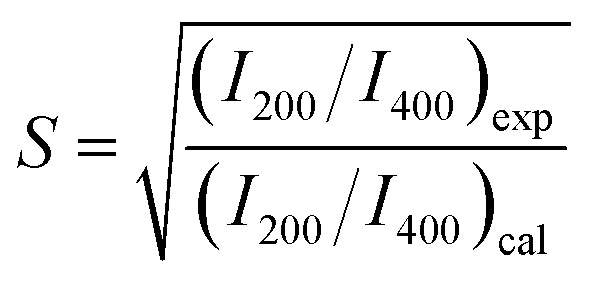
3
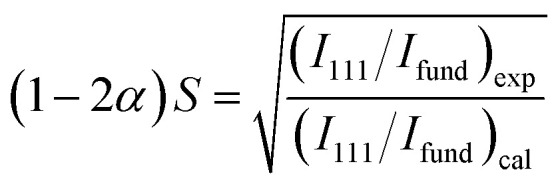
where *I*_200_, *I*_111_, and *I*_fund_ are integrated intensities of 200 and 111 superlattice peaks, and a fundamental peak, respectively, and “exp” and “cal” respectively, means an experimental value and a calculated one for the perfectly ordered case. *S* corresponds to the long-range order parameter for binary alloys,^28^ here describing the order between Co and Fe or Ge atoms (0 ≤ *S* ≤ 1); *α* describes the disorder between Fe and Ge atoms (0 ≤ *α* ≤ 0.5); thus, *S* = 1 with *α* = 0 means the perfect order. Although accurate evaluation of *I*_200_ and *I*_111_ was difficult because of too small an intensity, *S* and *α* were roughly estimated to be 0.8 and 0.0, respectively, when using the fitted data in Fig. 1c. Thus, the degree of long-range order was likely high.

**Fig. 1:**
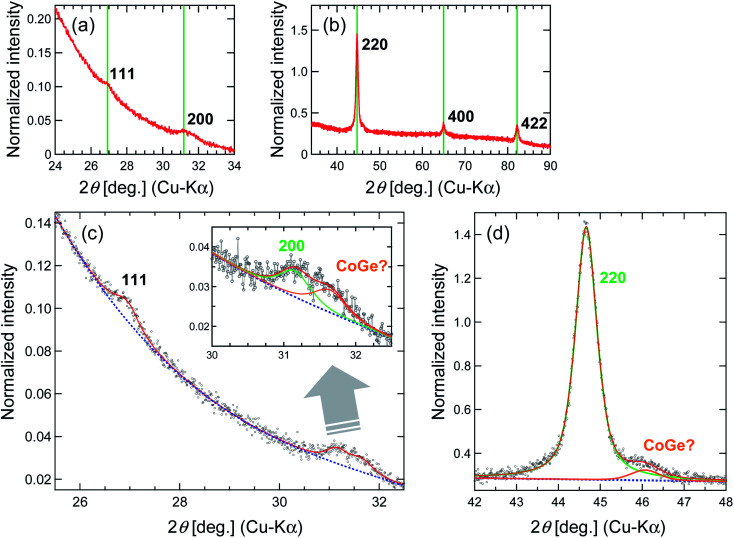
XRD patterns for Co_2_FeGe/SiO_2_ (a) around superlattice peaks, (b) around fundamental peaks, (c) around superlattice peaks with fitting, and (d) around 220 peak with fitting. All peaks were normalized by integrated intensity of 220 peak. Green vertical lines in (a and b) show peak positions observed for unsupported Co_2_FeGe powders. Blue dashed lines and red solid lines in (c and d) show backgrounds and sum of fitting lines, respectively. Inset of (c) displays magnification around 200 peak with fitting lines for 200 peak (green) and for second phase peak (orange) possibly originating from intermetallic CoGe phase, which also has a peak (orange) nearby 220 peak (green) in (d). Fitting was done using pseudo-Voigt function for peaks and polynomial function for backgrounds.

Fig. 2a shows the image obtained by high-resolution high-angle annular dark field scanning transmission electron microscopy (HAADF-STEM) (FEI Titan G2). Brighter particles with relatively uniform diameters below about 30 nm are observed on darker skeletal matter, which indicates that Co_2_FeGe nanoparticles are relatively homogeneously distributed on SiO_2_ supports. The diameters of the brighter particles were counted, as shown in Fig. 2b. The size distribution was relatively narrow. The volume-weighted average diameter (*d*_TEM_) was estimated to be 23.0 ± 5.3 nm by

4 *d*_TEM_ = Σ*n*_i_*d*_i_^4^/Σ*n*_i_*d*_i_^3^

where *n*_i_ is the number of particles with the diameter *d*_i_ in Fig. 2b.^20,21^Fig. 2c–f show elemental maps obtained by energy-dispersive X-ray (EDX) analysis. Co, Fe, and Ge were detected in the same regions, in which the brighter particles were observed in Fig. 2a. The quantitative analysis for the particle represented in Fig. 2g revealed that the chemical composition among Co, Fe, and Ge followed the precursor ratio and was close to the stoichiometry, as shown in Fig. 2h. These XRD and HAADF-STEM results clearly indicate the success of synthesizing Co_2_FeGe nanoparticles on SiO_2_ supports of sufficient quality.

**Fig. 2:**
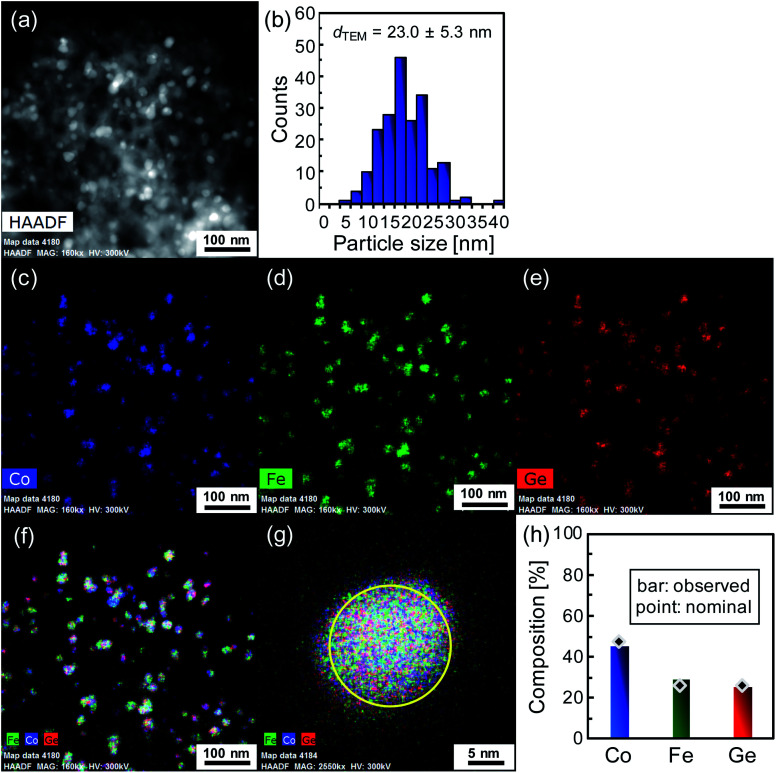
Analysis by HAADF-STEM with EDX for Co_2_FeGe/SiO_2_: (a) HAADF-STEM image, (b) histogram of diameter for bright particles in (a), elemental maps for (c) Co, (d) Fe, and (e) Ge, (f) superimposed elemental map, (g) magnified elemental map, and (h) chemical composition in area marked by yellow circle in (g). In (h) bars display observed values and points indicate nominal values estimated from precursor ratio.

The Co_2_FeGe/SiO_2_ was tested for catalytic reaction of the C_3_H_4_ hydrogenation using a standard flow reactor (see ref. 11 for details). Thirty mg of the catalyst was heated under H_2_ gas flow at 800 °C for 1 h to remove surface oxides; then, feeding of a gaseous mixture of [0.1% C_3_H_4_/40% H_2_/He balance] began at ambient temperature and pressure at 30 mL min^−1^ (20 °C, 0.1 MPa) (space velocity: about 40 000 h^−1^). The products were analyzed by gas chromatography (Agilent 490 Micro GC with PoraPLOT Q column) after waiting 30 min at each temperature. The conversion of C_3_H_4_ and the selectivity of products were estimated by5
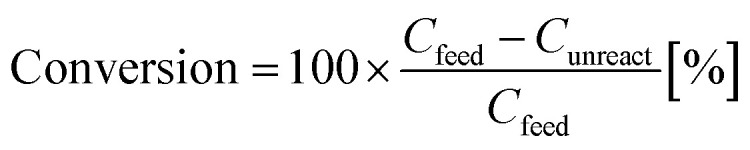
6
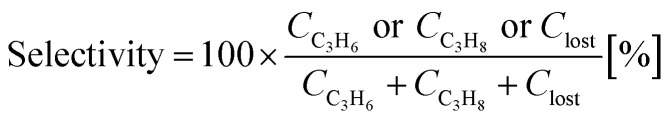
where *C*_feed_, *C*_unreact_, *C*_C_3_H_6__, and *C*_C_3_H_8__ were the concentrations of the feed C_3_H_4_, the unreacted C_3_H_4_, the produced C_3_H_6_, and the produced C_3_H_8_, respectively. *C*_lost_ is the concentration of carbon species lost due to oligomerization or coking, which is estimated by *C*_lost_ = *C*_feed_ − *C*_unreact_ − *C*_C_3_H_6__ − *C*_C_3_H_8__.

Fig. 3a shows the results of the catalytic test. The carbon lost was negligible. The C_3_H_6_ selectivity was as high as over 70% even when the C_3_H_4_ conversion was 100%. In general, strong adsorption of C_3_H_4_ prevents re-adsorption of C_3_H_6_, which suppresses the further hydrogenation of C_3_H_6_, resulting in high C_3_H_6_ selectivity when the C_3_H_4_ conversion is below 100%. Once all C_3_H_4_ is consumed, C_3_H_6_ is quickly hydrogenated; thus, the C_3_H_6_ selectivity drastically decreases once the C_3_H_4_ conversion achieves 100% in most catalysts, including pure metals^29,30^ and Co_2_FeGa^11^ (Fig. S1a and b†). Therefore, the Co_2_FeGe/SiO_2_ synthesized here has an intrinsic selectivity for C_3_H_6_ as well as the unsupported Co_2_FeGe powders synthesized metallurgically, the C_3_H_6_ selectivity of which was over 90% even when the C_3_H_4_ conversion was 100% (Fig. S2†).^11^ The reaction rate per weight of Co used was significantly enhanced up to as much as 2000 times by nano-sizing compared with the unsupported one, as shown in Fig. 3b. In terms of stability, a small deactivation was observed in the cooling process after heating up to 200 °C (Fig. S3†), likely due to oligomerization or coking, while the C_3_H_6_ selectivity was improved over 90%.

**Fig. 3:**
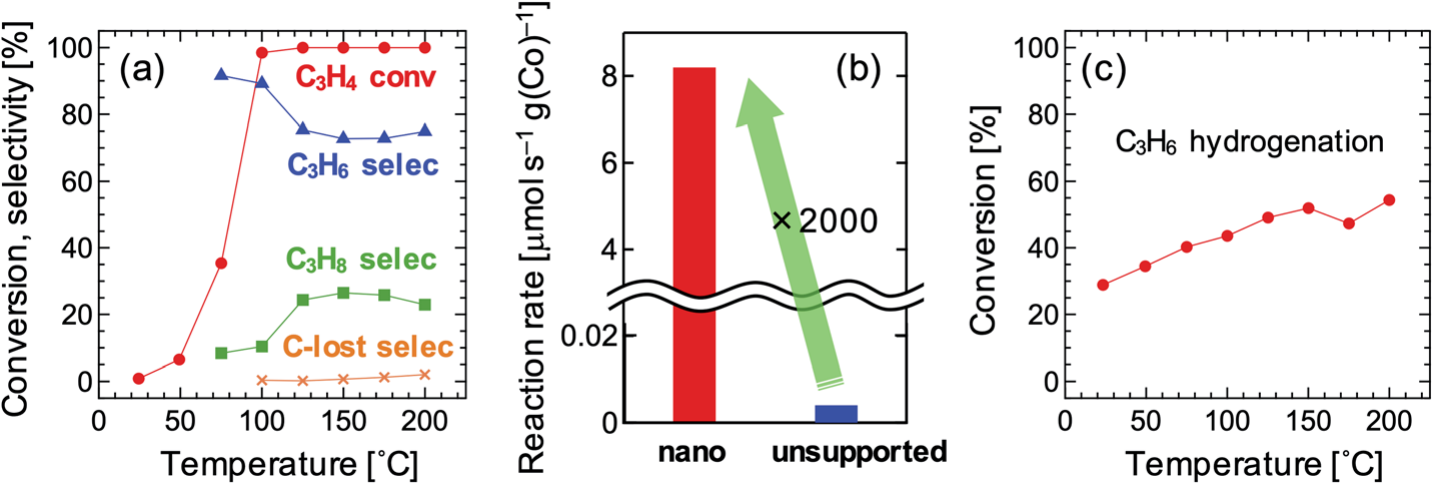
(a) Catalytic properties of Co_2_FeGe/SiO_2_ for C_3_H_4_ hydrogenation, (b) C_3_H_4_ reaction rate per weight of Co at 75 °C for Co_2_FeGe/SiO_2_ (“nano”) and unsupported Co_2_FeGe (“unsupported”), (c) C_3_H_6_ conversion for Co_2_FeGe/SiO_2_ in C_3_H_6_ hydrogenation. Reaction conditions in (c) were the same as those in (a) except the reactant, which was [0.1% C_3_H_6_/40% H_2_/He-balance] in (c).

To reveal the reason for the lower C_3_H_6_ selectivity of the Co_2_FeGe/SiO_2_ than that of the unsupported Co_2_FeGe, the catalytic test for C_3_H_6_ hydrogenation was conducted in the same manner as the C_3_H_4_ hydrogenation as shown in Fig. 3c. A certain amount of C_3_H_6_ was converted to C_3_H_8,_ whereas it was scarcely converted by the unsupported one.^11^ This means the presence of the sites that further hydrogenate C_3_H_6_ in the C_3_H_4_ hydrogenation. For ordinary catalysts, the conversion is larger for the C_3_H_6_ hydrogenation than C_3_H_4_ hydrogenation at a lower temperature region in these reaction conditions^11^ (Fig. S1†). The larger conversion of C_3_H_6_ than C_3_H_4_ at ≤75 °C for the Co_2_FeGe/SiO_2_ (Fig. 3a and c) thus also indicates the presence of non-selective sites for the C_3_H_4_ hydrogenation. An anomaly at 175 °C in Fig. 3c is likely a result of conflict between the acceleration of reaction and the deceleration of C_3_H_6_ adsorption along with increasing temperature, which is often observed.^11^

Taking into account the origin of the high alkene selectivity that inactive Ge atoms shrink the size of active ensembles and thereby prevent the re-adsorption of alkene molecules, which is indicated from electronic structures,^11^ two candidates are considered for the non-selective sites. One is a monometallic Co ensemble. In this impregnation synthesis, a part of the particles likely have chemical compositions that deviate from the target value. In particles with excess Co, monometallic Co ensembles should form, which is active for hydrogenation but not selective, as indicated by the tests using Co/SiO_2_ (Fig. S1a and c†). Actually, Co_2_FeGe/SiO_2_ catalysts preliminary prepared with the atomic ratio of Co : Fe: Ge = 2 : 1 : 1 loaded exhibited a poor selectivity (Fig. S4a†) in contrast to the present catalyst (loaded Co : Fe : Ge = 1.8 : 1 : 1), and the former showed an extra peak in the temperature programmed CO desorption profile as well as the pure Co in addition to the peaks for the unsupported Co_2_FeGe (Fig. S4b†).^31^ The other candidate for non-selective sites is a specific site formed by nano-sizing, such as the corner and the edge. These low-coordinated sites are generally active but in different environments from the terrace sites; thus, they can be non-selective. A tiny amount of the second phase CoGe is unlikely as the candidate because a high ethylene selectivity in selective hydrogenation of acetylene by CoGe has been reported.^32^

Although it cannot be concluded whether the Co ensembles or the specific sites dominated the reduction of selectivity, the selectivity will increase up to the value for the unsupported one if the non-selective sites are identified and removed. Actually, the C_3_H_6_ selectivity was improved along with the deactivation (Fig. S3†), indicating that the non-selective sites were killed by carbon deposition due to oligomerization or coking. Nevertheless, over 70% for C_3_H_6_ selectivity at 100% conversion of C_3_H_4_ is high enough for the condition under abundant hydrogen (C_3_H_4_ : H_2_ = 1 : 400). This high selectivity was also confirmed in the C_3_H_4_ hydrogenation in the presence of abundant C_3_H_6_ (Fig. S5†). These results indicate that the excellent catalytic properties of intermetallic micro-powders can be conserved in their nanoparticles. The reaction rate of C_3_H_4_ per surface area of Co_2_FeGe was roughly estimated to be 1.2 × 10^−7^ mol s^−1^ m^−2^ at 75 °C by assuming the 23 nm-spheres with density of 8.66 g cm^−3^ (estimated by XRD for the unsupported one and using atomic weights). The value for the unsupported one was 4.1 × 10^−8^ mol s^−1^ m^−2^ at 75 °C. Although the estimation was very approximate, it can at least be said that the reaction rate did not decrease, or rather, it seems that the reaction rate somewhat increased by nano-sizing. This fact also assures the conservation of intrinsic catalytic properties after nano-sizing, while which increases the surface energy, enhancing the adsorption of reactant species, thereby possibly increasing the reaction rate.

The catalytic performance is compared with those of other supported intermetallic catalysts reported (Table 1). Since Ni is a typical catalyst for hydrogenation, 3d-transition-metal-based intermetallic catalysts reported for selective hydrogenation of alkynes are mostly Ni-based. Although the performance cannot be exactly compared, because the reported catalysts were tested for selective hydrogenation of acetylene (C_2_H_2_) with different reaction conditions, the selectivity of Co_2_FeGe/SiO_2_ seems to be at the same level as those of the reported catalysts. By comparing a specific reaction rate per weight of Ni or Co at 100 °C, the activity of Co_2_FeGe/SiO_2_ also seems to be at the same level as those of the reported catalysts.

**Table 1:** Catalytic performance of supported intermetallic catalysts for selective hydrogenation of alkynes. C_2_H_2_/C_2_H_4_ was used as a reactant in literature

Catalyst	Ni(Co) wt%	Amount [mg]	C_2_H_2_(C_3_H_4_) flow rate [mL min^−1^]	C_2_H_2_(C_3_H_4_) : H_2_ : C_2_H_4_(C_3_H_6_) : He(Ar, N_2_)	GHSV [mL g^−1^ h^−1^]	Conv. [%]	Selec. [%]	Temp. [°C]	Specific rate [mL_C_2_H_2_(C_3_H_4_)_ min^−1^ g_Ni(Co)_^−1^]	Ref.
Ni_3_Ge/MCM-41	3.2	16	3.9	3.9 : 8 : 0 : 17.1	107520	30	85	250	2340	33
NiGa/Mg/Al-LDH	10	50	1.2	1.2 : 12 : 0 : 106.8	144000	94	81	220	226	34
						26	82	100	62	
			1.2	1.2 : 12 : 24 : 82.8	144000	72	75	186	173	
						7	87	93	17	
Ni_3_Ga/MgAl_2_O_4_	2	100	0.33	0.33 : 6.7 : 33.3 : 26.67	40 000	91	77	200	152	35
						9	96	100	15	
Co_2_FeGe/SiO_2_	3	30	0.03	0.03 : 12 : 0:17.97	60 000	98	89	100	33	This work
	3	60	0.03	0.03 : 12 : 3 : 14.97	30 000	98	77	100	16	

## Conclusions

We have successfully synthesized the SiO_2_ supported Co_2_FeGe nanoparticles by the co-impregnation method. The XRD indicated that the sample was almost a single phase of a highly ordered Heusler structure. The HAADF-STEM with EDX indicated that Co_2_FeGe nanoparticles with the average size of 23 nm were relatively homogeneously distributed on SiO_2_ supports. This nano-sizing enhanced the reaction rate per weight for C_3_H_4_ hydrogenation by 2000 times compared with the unsupported powders, while conserving high C_3_H_6_ selectivity. This study proves that even ternary intermetallic compounds can be downsized into supported nanoparticles with conserving intrinsic catalytic properties. In future, supported nanoparticles of various ternary intermetallic compounds including Heusler alloys would be developed not only as catalysts but also as other functional materials.

## Conflicts of interest

There are no conflicts to declare.

## Supplementary Material

RA-011-D1RA02884G-s001

## References

[cit1] Tsai A. P., Kameoka S., Ishii Y. (2004). J. Phys. Soc. Jpn..

[cit2] Armbrüster M., Schlögl R., Grin Y. (2014). Sci. Technol. Adv. Mater..

[cit3] Tsai A. P., Kameoka S., Nozawa K., Shimoda M., Ishii Y. (2017). Acc. Chem. Res..

[cit4] Furukawa S., Komatsu T. (2017). ACS Catal..

[cit5] Armbrüster M. (2020). Sci. Technol. Adv. Mater..

[cit6] Furukawa S., Komatsu T., Shimizu K. (2020). J. Mater. Chem. A.

[cit7] Dshemuchadse J., Steurer W. (2015). Acta Crystallogr..

[cit8] Gong Y., Wu J., Kitano M., Wang J., Ye T. N., Li J., Kobayashi Y., Kishida K., Abe H., Niwa Y., Yang H., Tada T., Hosono H. (2018). Nat. Catal..

[cit9] Wu J., Li J., Gong Y., Kitano M., Inoshita T., Hosono H. (2019). Angew. Chem., Int. Ed..

[cit10] Kojima T., Kameoka S., Tsai A.-P. (2017). ACS Omega.

[cit11] Kojima T., Kameoka S., Fujii S., Ueda S., Tsai A.-P. (2018). Sci. Adv..

[cit12] Kojima T., Kameoka S., Tsai A.-P. (2019). Sci. Technol. Adv. Mater..

[cit13] Kojima T., Kameoka S., Tsai A.-P. (2019). ACS Omega.

[cit14] Kojima T., Kameoka S., Tsai A.-P. (2021). KONA Powder Part. J..

[cit15] Basit L., Wang C., Jenkins C. A., Balke B., Ksenofontov V., Fecher G. H., Felser C., Mugnaioli E., Kolb U., Nepijko S. A., Schönhense G., Klimenkov M. (2009). J. Phys. D: Appl. Phys..

[cit16] Wang C., Basit L., Khalavka Y., Guo Y., Casper F., Gasi T., Ksenofontov V., Balke B., Fecher G. H., Sönnichsen C., Hwu Y. K., Lee J. J., Felser C. (2010). Chem. Mater..

[cit17] Wang C. H., Guo Y. Z., Casper F., Balke B., Fecher G. H., Felser C., Hwu Y. (2010). Appl. Phys. Lett..

[cit18] Wang C., Casper F., Guo Y., Gasi T., Ksenofontov V., Balke B., Fecher G. H., Felser C., Hwu Y. K., Lee J. J. (2012). J. Appl. Phys..

[cit19] Ernst S. , Malter O., Schuessler A., Braunsmann K., Trukhan N. and Mueller U., inventor; BASF, SE, assignee, *US Pat.*, US 2019/0358613 A1, 2019

[cit20] Nakaya Y., Miyazaki M., Yamazoe S., Shimizu K., Furukawa S. (2020). ACS Catal..

[cit21] Nakaya Y., Hirayama J., Yamazoe S., Shimizu K., Furukawa S. (2020). Nat. Commun..

[cit22] Ida T., Shimazaki S., Hibino H., Toraya H. (2003). J. Appl. Crystallogr..

[cit23] Sasaki S. , KEK Report, 1989, 88–14, pp. 1–136, calculated values can be downloaded from http://www.sasakiken.net/indexe.html

[cit24] Butt N. M., Bashir J. (1988). Acta Crystallogr., Sect. A: Found. Crystallogr..

[cit25] International Tables for Crystallography Vol C Mathematical, Physical and Chemical Tables, ed. E. Prince, Kluwer Academic Publishers, 3rd edn, London, 2004, p. 239

[cit26] Kojima T., Kameoka S., Tsai A.-P. (2018). ACS Omega.

[cit27] Webster P. J., Ziebeck K. R. A. (1973). J. Phys. Chem. Solids.

[cit28] Warren B. E. , X-Ray Diffraction, Dover Publications, New York, 1990, p. 208

[cit29] Yoshida N. , PhD thesis, Osaka University, Japan, 1971

[cit30] Kojima T., Fujieda S., Kato G., Kameoka S., Suzuki S., Tsai A. P. (2017). Mater. Trans..

[cit31] Kojima T. , Koganezaki T., Fujii S., Kameoka S. and Tsai A.-P., Catal. Sci. Technol., 2021, 10.1039/D1CY00279A

[cit32] Komatsu T., Fukui M., Yashima T. (1996). Stud. Surf. Sci. Catal..

[cit33] Komatsu T., Kishi T., Gorai T. (2008). J. Catal..

[cit34] Cao Y., Zhang H., Ji S., Sui Z., Jiang Z., Wang D., Zaera F., Zhou X., Duan X., Li Y. (2020). Angew. Chem., Int. Ed..

[cit35] Liu Y., Liu X., Feng Q., He D., Zhang L., Lian C., Shen R., Zhao G., Ji Y., Wang D., Zhou G., Li Y. (2016). Adv. Mater..

