# Supplementary material for: Synthesis of Co2FeGe Heusler alloy nanoparticles and catalysis for selective hydrogenation of propyne
Source: RSC Adv. 2021 May 19;11(29):18074–9. doi: 10.1039/d1ra02884g (PMC9033194; doi:10.1039/d1ra02884g)
Supplement: RA-011-D1RA02884G-s001 [file RA-011-D1RA02884G-s001.pdf]

Electronic Supplementary Material (ESI) for RSC Advances.
This journal is © The Royal Society of Chemistry 2021

Electronic Supplementary Information

Synthesis of Co_2FeGe Heusler alloy nanoparticles and catalysis for selective hydrogenation of propyne

Takayuki Kojima,*[a,b,†] Yuki Nakaya,[c] Hyugwon Ham,[c]
Satoshi Kameoka,[b] Shinya Furukawa*[c]

[a] Frontier Research Institute for Interdisciplinary Sciences, Tohoku University
Sendai, Japan

[b] Institute of Multidisciplinary Research for Advanced Materials, Tohoku University,
Sendai, Japan

[c] Institute for Catalysis, Hokkaido University, Sapporo, Japan

† Present address: Division of Chemistry and Materials, Faculty of Textile Science
and Technology, Shinshu University, Ueda, Japan

* Corresponding authors
TK: tkojima@shinshu-u.ac.jp
SF: furukawa@cat.hokudai.ac.jp

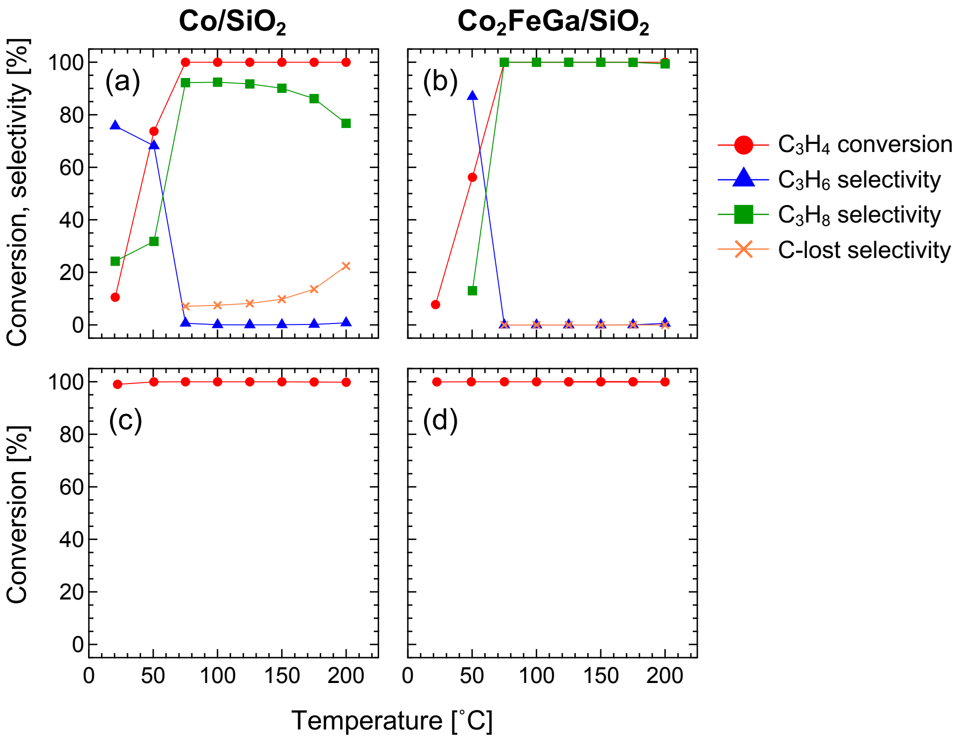

Fig. S1. Results of (a,b) C_3H_4 hydrogenation and (c,d) C_3H_6 hydrogenation for (a,c) Co/SiO_2 and (b,d) Co_2FeGa/SiO_2. Legends at the right side of (b) are available for (a,b). The weight of catalysts used was 10 mg for all tests. All other reaction conditions were the same as those for Co_2FeGe/SiO_2. These catalysts were synthesized in the same manner as Co_2FeGe/SiO_2 except that $Ga(NO_3)\cdot 8H_2O$ was used and the Co weight was 3.5 wt% for Co_2FeGa/SiO_2.

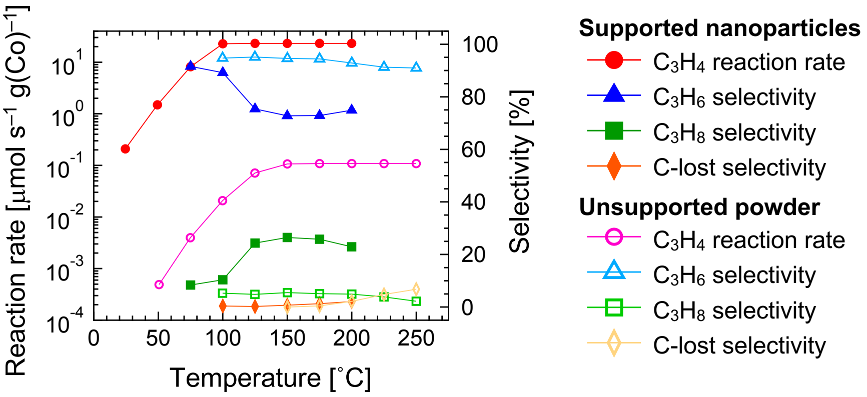

Fig. S2. Comparison between Co_2FeGe/SiO_2 and unsupported Co_2FeGe for C_3H_4 hydrogenation. Plateau for reaction rate corresponds to 100% of conversion. Data for unsupported Co_2FeGe was excerpted from Ref. [S1] licensed under CC BY-NC (http://creativecommons.org/licenses/by-nc/4.0).

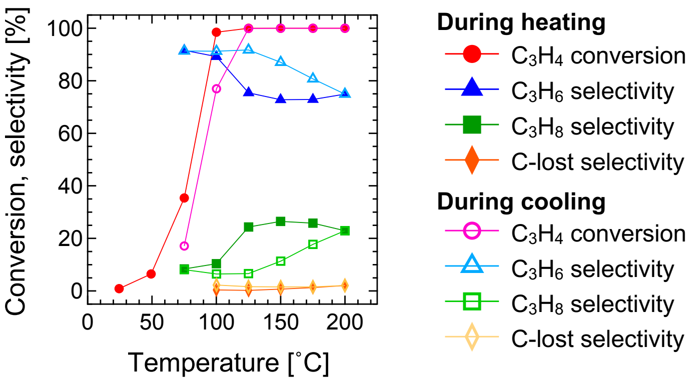

Fig. S3. Catalytic properties of Co_2FeGe/SiO_2 for C_3H_4 hydrogenation during heating and cooling cycle.

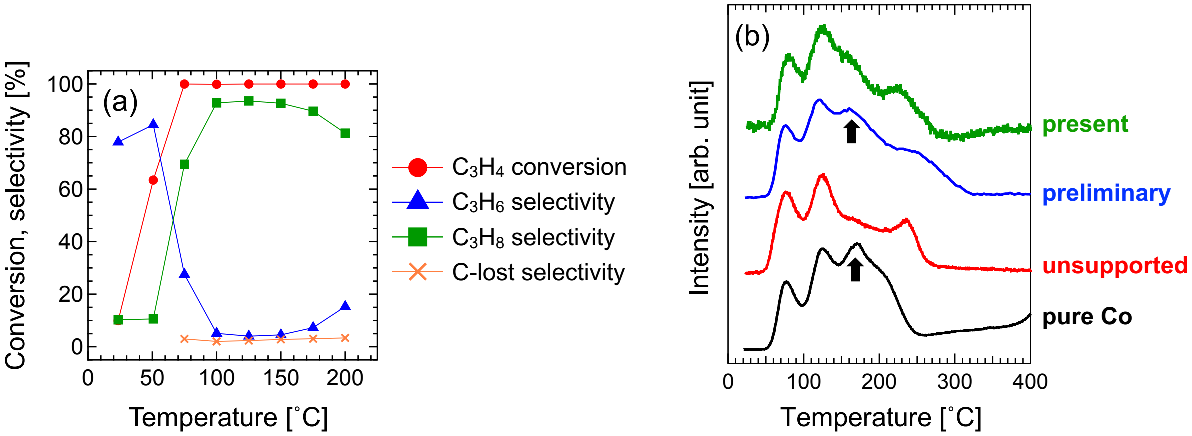

Fig. S4. (a) Example of preliminary results of C_3H_4 hydrogenation for Co_2FeGe/SiO_2 prepared with loading atomic ratio of Co:Fe:Ge = 2:1:1 and (b) profiles of temperature programmed CO desorption measured by mass spectroscopy for present Co_2FeGe/SiO_2 (loaded Co:Fe:Ge = 1.8:1:1), preliminary Co_2FeGe/SiO_2, unsupported Co_2FeGe, and unsupported pure Co. In (b), a specific peak likely originated from the non-selective active sites is indicated by arrow, and data for unsupported Co_2FeGe and pure Co were reproduced from Ref. [S2] with permission from The Royal Society of Chemistry.

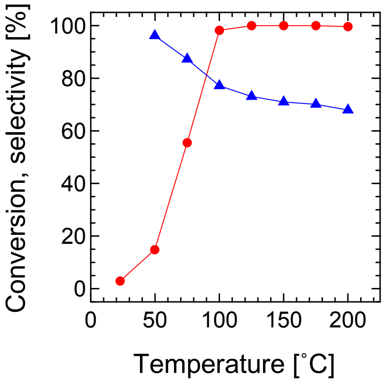

$$C_3H_6\ selectivity = 100 \times \frac{C_{\mathrm{feed}} - C_{\mathrm{unreact}}}{C_{\mathrm{feed}} - C_{\mathrm{unreact}} + C_{\mathrm{C3H8}}}\ [\%]$$

Fig. S5. Catalytic properties of Co_2FeGe/SiO_2 for selective hydrogenation of C_3H_4 in the presence of abundant C_3H_6 using a reactant of [0.1%C_3H_4 / 10%C_3H_6 / 40%H_2 / He-balance]. The C_3H_6 selectivity was evaluated by the displayed equation [S1], where C_{feed}, C_{unreact}, and C_{C3H8} were the concentrations of the feed C_3H_4, the unreacted C_3H_4, and the produced C_3H_8, respectively. The weight of catalyst used was 60 mg. All other reaction conditions were the same as those in other reactions.

References

S1. T. Kojima, S. Kameoka, S. Fujii, S. Ueda and A.-P. Tsai, *Sci. Adv.*, 2018, **4**, eaat6063.

S2. T. Kojima, T. Koganezaki, S. Fujii, S. Kameoka, and A.-P. Tsai, *Catal. Sci. Technol.*, 2021 (in press). DOI: 10.1039/D1CY00279A
